# Distortion in LPBF Cantilevers Governed by Stiffness-Controlled Stress Redistribution

**DOI:** 10.3390/ma19163407

**Published:** 2026-08-11

**Authors:** Yunpeng Zhang, Xiaojiong Nie, Xin Liao, Xin Lin, Xufei Lu

**Affiliations:** 1 State Key Laboratory of Solidification Processing, Northwestern Polytechnical University, Xi’an 710072, Chinaniexj@mail.nwpu.edu.cn (X.N.);; 2 MIIT Key Laboratory of Metal High Performance Additive Manufacturing and Innovative Design, Northwestern Polytechnical University, Xi’an 710072, China

**Keywords:** laser powder bed fusion, cantilever structure, stress-induced distortion, structural stiffness, thermo-mechanical simulation

## Abstract

Residual stresses generated during laser powder bed fusion (LPBF) can cause substantial distortion in slender structures, compromising dimensional accuracy and structural reliability. This study tests the hypothesis that, under identical nominal processing conditions, geometry-dependent stiffness and constraint govern how the evolving thermally induced stress field is redistributed and manifested as warpage after support removal. Bridge-type TA15 titanium alloy cantilever specimens with different spans, thicknesses, and support densities were fabricated by LPBF, and their post-cut warpage was quantified by three-dimensional scanning. A coupled thermo-mechanical finite element model was validated against the measured deformation profiles and subsequently used to examine simulated stress evolution during deposition and redistribution after support removal. Cantilevers with different spans approached similarly high simulated surface tensile-stress plateaus in the constrained as-built state but exhibited markedly different measured warpage after cutting, showing that the as-built stress magnitude alone does not reliably rank post-release deformation. Increasing span reduced global flexural resistance and enlarged the effective bending arm, whereas increasing thickness enhanced flexural rigidity and suppressed curvature even when relatively high localized stress was retained. With the total support volume held constant, changing support density altered the system-level constraint through the combined effects of support-leg stiffness, support spacing, local thermal and mechanical response, and deformation compatibility. Together, these results provide an experimentally supported process-structure interpretation of LPBF cantilever distortion across controlled variations in span, thickness, and support distribution.

## 1. Introduction

Laser powder bed fusion (LPBF) has become an important manufacturing route for titanium alloy components with complex geometries and high structural efficiency, and has found wide application in aerospace, biomedicine, and other high-end engineering fields [1,2]. As a key enabling technology for lightweight design and customized manufacturing, LPBF offers unique advantages in producing geometrically complex parts that are difficult to fabricate by conventional methods [3]. However, localized melting, rapid solidification, and repeated thermal cycling generate steep temperature gradients and thermally induced strain mismatch, thereby promoting the accumulation of residual stress [4,5,6,7]. Such residual stresses are widely recognized as a major cause of dimensional inaccuracy, support failure, and post-fabrication distortion in LPBF components [8,9].

Among the various structural forms encountered in LPBF, cantilever-type geometries are particularly susceptible to deformation [10,11]. These features are commonly found in overhanging transition regions, thin-walled extensions, and local protruding sections. During fabrication, the cantilever is constrained by the support structure and the substrate, so thermal strain cannot be freely released and residual stress gradually accumulates within the suspended region [12,13,14]. Once the supports are removed or the part is separated from the substrate, the stored stress is redistributed and often manifests as noticeable bending or warpage [15,16,17]. In practice, marked differences in distortion may occur even under identical nominal processing conditions [18,19], indicating that geometry-dependent thermal and mechanical responses also contribute to the final deformation.

For slender cantilever structures, this response is particularly sensitive to span and sectional stiffness [20,21,22]. Increasing the cantilever length enlarges the effective bending arm and tends to amplify deformation under a comparable internal-stress state [23,24], whereas increasing thickness raises the sectional moment of inertia and flexural rigidity, thereby suppressing bending [11,25,26,27]. Span and thickness therefore provide controlled geometric variables for examining how thermally induced stress redistribution is manifested as post-release deformation.

Support configuration introduces an additional level of complexity. Supports provide temporary mechanical restraint and thermal conduction paths during LPBF, thereby affecting stress accumulation during deposition and redistribution after removal [9,17,28]. When the total support volume is unchanged, increasing the number of support legs produces a more spatially continuous constraint while reducing the cross-sectional stiffness of each individual leg. Conversely, coarse supports provide stronger local restraint but create larger unsupported intervals. The resulting deformation therefore depends on the combined effects of support-leg stiffness, support spacing, local thermal and mechanical response, and deformation compatibility rather than on support density alone.

Previous LPBF studies relevant to distortion can be broadly divided into three lines of investigation. Process- and melt-pool-scale studies have examined the effects of laser parameters, heat-source characteristics, melt-pool evolution, and thermal history on defect formation and material response [29,30,31,32,33,34]. Thermo-mechanical and distortion-prediction studies have investigated temperature gradients, residual-stress development, material properties, geometric dimensions, and final deformation [8,9,12,13,14,15,16,17,18,19]. Support-related studies have focused on support strength, manufacturability, material consumption, removal effort, and equivalent or apparent mechanical properties [17,18,28,35,36,37]. These approaches have established the importance of thermal loading, geometry, and support conditions in LPBF distortion, but they differ substantially in modeling scale, research objective, and treatment of structural constraint.

These existing approaches are appropriate for their respective objectives but do not always isolate the specific process-structure relationship considered here. Process- and melt-pool-scale models are primarily intended to resolve localized thermal and material responses rather than structural deformation after support removal. Structural-scale distortion models often emphasize the final stress or deformation field and may represent support regions using simplified geometries or homogenized properties, limiting the direct separation of support-leg stiffness, spacing, and system-level constraint. Moreover, support-density studies frequently vary the total support volume together with the number and spacing of support units, making it difficult to distinguish the influence of support distribution from that of the amount of support material. Consequently, relatively few studies have combined controlled variations in cantilever span, sectional thickness, and support distribution under identical nominal processing conditions with measured post-cut warpage and simulated stress evolution both before and after support removal.

To address this specific gap, the present study tests the hypothesis that, under identical nominal LPBF processing conditions, geometry-dependent thermal history, structural stiffness, and constraint govern how the thermally induced stress field is redistributed and manifested as warpage after support removal. Accordingly, the as-built or post-removal residual-stress magnitude alone may not reliably rank the final deformation of cantilevers with different geometries or support configurations. Cantilever specimens with different spans, sectional thicknesses, and support distributions were fabricated under controlled conditions, and their post-cut warpage was quantified by three-dimensional scanning. For the support comparison, the total support volume was held constant to distinguish the influence of support distribution from that of the amount of support material.

The hypothesis was examined by combining experimentally measured post-cut warpage with a deformation-validated thermo-mechanical finite element model. The model was used to examine longitudinal-stress evolution at selected build heights and along characteristic internal and top-surface paths before and after support removal. The specific contribution is a controlled process-structure comparison that relates measured deformation to simulation-resolved stress evolution across variations in cantilever span, sectional thickness, and support distribution. The analysis focuses on whether comparable simulated as-built surface-stress levels can correspond to different post-cut warpage and whether relatively high localized stress can coexist with limited curvature in structurally stiff cantilevers.

## 2. Experimental Procedure

### 2.1. Materials and LPBF Experiments

To investigate the thermo-mechanical response of LPBF-fabricated cantilever structures with different geometric features, a series of bridge-type specimens were fabricated using TA15 titanium alloy powder. An annealed TC4 titanium alloy plate with dimensions of 250 × 250 × 20 mm^3^ was used as the substrate. Before fabrication, the substrate surface was mechanically ground to remove the native oxide layer and obtain a uniform surface condition, followed by ultrasonic cleaning in acetone to eliminate organic contaminants. This treatment helped ensure stable metallurgical bonding and consistent interfacial heat transfer during the LPBF process.

**Figure 1:**
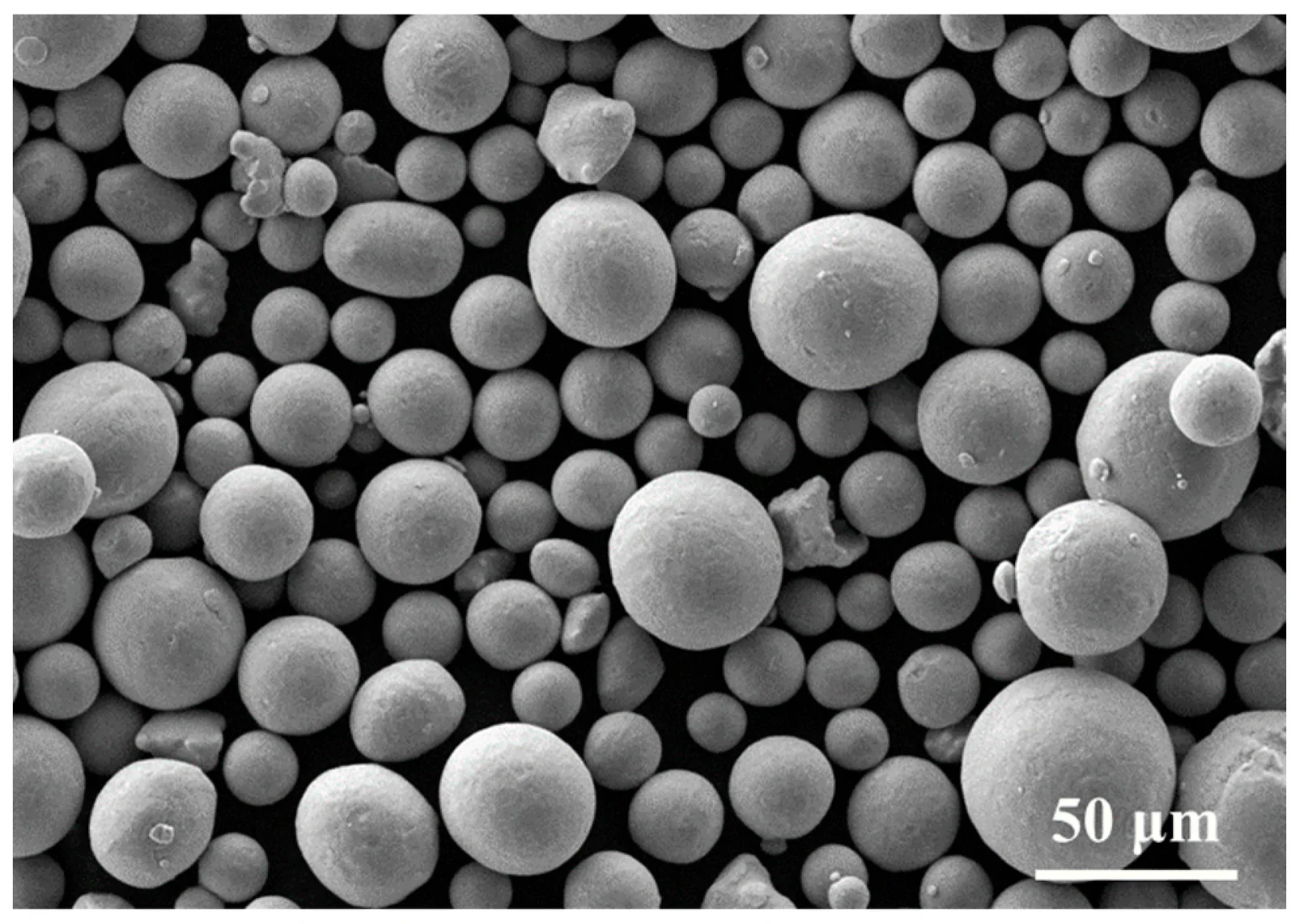
Surface morphology of the TA15 powder used in LPBF.

The feedstock material was TA15 titanium alloy powder prepared by electrode induction melting inert gas atomization. The powder particle size ranged from 15 to 53 μm, and its chemical composition is listed in Table 1. As shown in Figure 1, the powder particles are predominantly spherical, with only a small number of satellite particles, indicating good flowability and spreading behavior. The measured Hall flow rate, apparent density, and tap density were 34.4 g/50 s, 2.42 g/cm^3^, and 2.81 g/cm^3^, respectively. Before processing, the powder was vacuum-dried at 120 °C for 3 h to reduce moisture absorption and minimize the risk of porosity and hydrogen pickup during fabrication.

**Figure 2:**
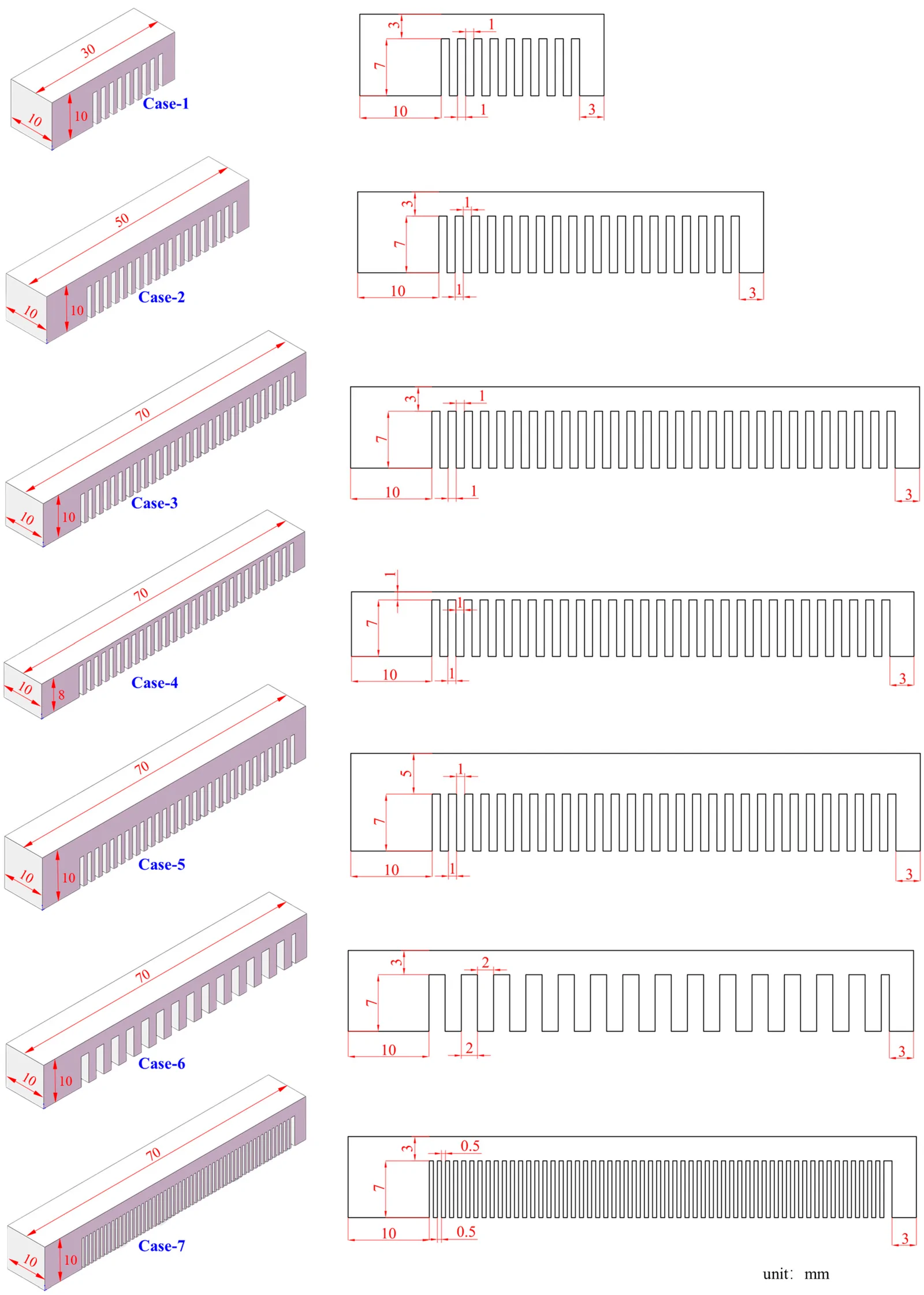
Schematic diagram of the cantilever geometry fabricated by LPBF.

Seven cantilever structures with different geometric configurations were designed, as illustrated in Figure 2. The unsupported span, cantilever thickness, and support distribution were selected as the main geometric variables, so as to generate different structural stiffnesses, thermal responses, and constraint conditions during printing. Among them, Cases 1–3 were used to evaluate the effect of cantilever span, Cases 3–5 to examine the effect of cantilever thickness, and Cases 3, 6, and 7 to investigate the effect of support density under a constant total support volume.

The specimens were fabricated using a BLT-A300 LPBF system (Xi’an Bright Laser Technologies Co., Ltd., Xi’an, Shaanxi, China) equipped with a 500 W Yb-fiber laser. All experiments were carried out in a high-purity argon atmosphere, with the oxygen content maintained below 100 ppm to suppress oxidation. The processing parameters are summarized in Table 2. No substrate preheating was applied, so that steep thermal gradients and the associated residual stress accumulation could develop during the building process.

An alternating scanning strategy with a 67° rotation between successive layers was adopted to reduce directional bias in heat accumulation and stress development. The laser scanning followed a stripe-based filling mode with a stripe width of 10 mm and an overlap of 0.02 mm between adjacent stripes, so as to ensure sufficient remelting and bonding between neighboring scan tracks. After fabrication, the loose powder was removed, and all specimens were kept in the as-built state prior to the subsequent stress-release operation.

No separate skin, up-skin, down-skin, or contour parameter sets were used. The same processing parameters listed in Table 2 were applied to all bulk, surface, and contour regions for all seven specimen configurations.

### 2.2. Support Removal and Warpage Characterization

**Figure 3:**
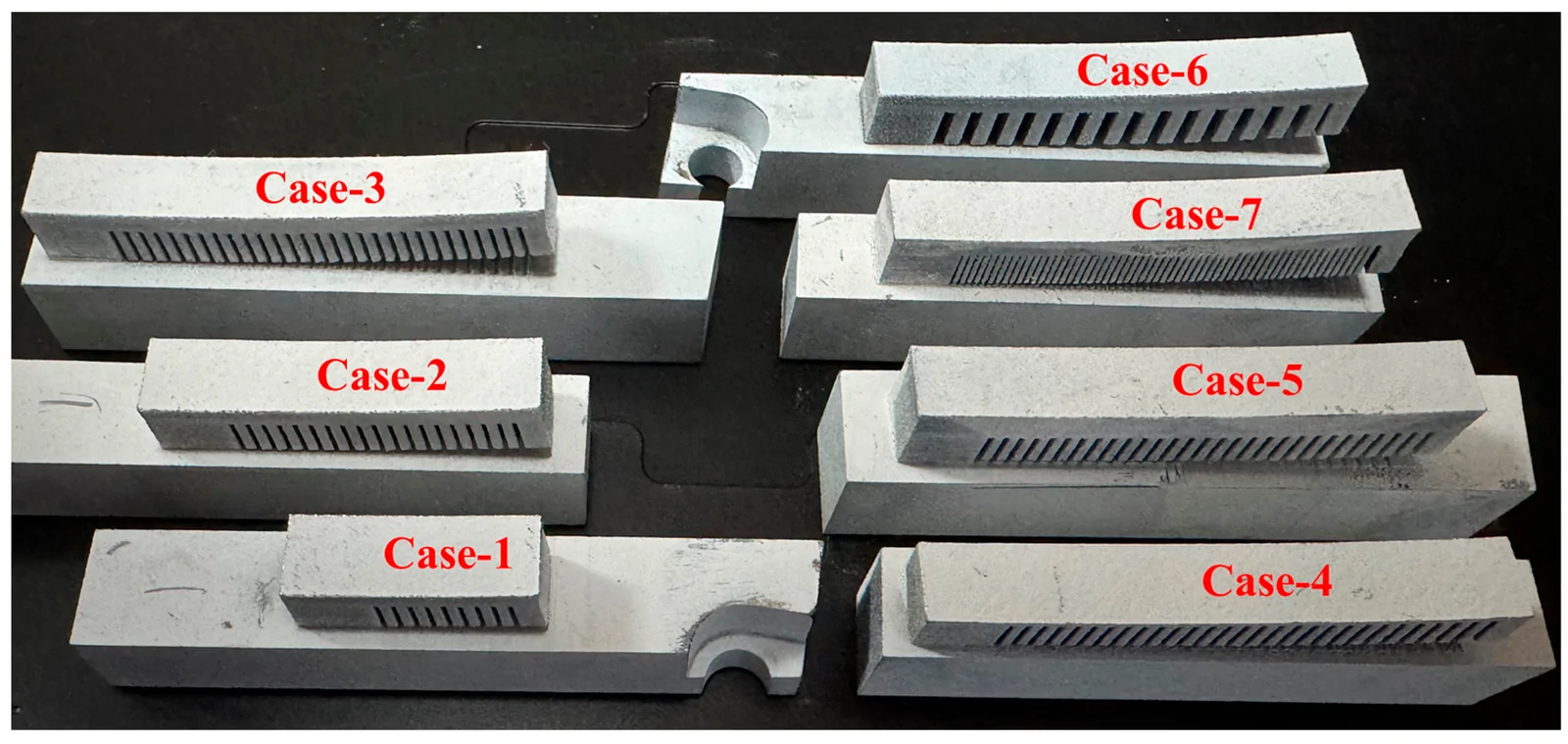
LPBF-fabricated cantilever structures after support removal.

To evaluate the deformation induced by constraint release, the supports were separated from the substrate by wire electrical discharge machining (WEDM) along the support-substrate interface. For all configurations, cutting started from the free-end side, and all support legs beneath the suspended region were severed while the 10 mm long solid root anchoring block remained connected to the substrate. Because the cantilever spans differed, the total nominal cutting distance was 20 mm for Case 1, 40 mm for Case 2, and 60 mm for Cases 3–7. Thus, the cutting distance was not identical, but the nominal constraint-release procedure and retained-root condition were consistent among the configurations. This controlled operation released the support constraint, allowing the internal stress field to redistribute and the resulting out-of-plane bending to develop, as shown in Figure 3.

The retained-end anchoring conditions were controlled to be nominally comparable across the seven configurations. All specimens used the same 10 mm × 10 mm × 7 mm solid anchoring block, support–substrate connection geometry, substrate material, surface preparation, protective atmosphere, layer thickness, processing parameters, scan strategy, and WEDM datum. The specimens were fabricated in the same build batch, and no interfacial delamination or support failure was observed before cutting. These controls provided comparable retained-end constraint conditions among the tested configurations. However, the interfacial adhesion strength was not independently measured.

The three-dimensional deformation field of each specimen after cutting was measured using a Lingchuang G5-630 industrial 3D scanner (Guangzhou Flash Creation 3D Technology Co., Ltd., Guangzhou, Guangdong, China), with a stated accuracy of ±0.01 mm over 600 mm. The scanned geometries were aligned with the corresponding nominal CAD models in *Geomagic Control X* (v2022.1), and the vertical displacement distribution along the top surface was extracted to quantify the warpage magnitude and spatial variation in each cantilever.

One specimen was fabricated for each cantilever configuration, and each specimen was subjected to three repeated 3D scans to assess measurement repeatability. At each measurement position, the absolute deviation of each scan from the three-scan mean was calculated. The mean absolute variation over the evaluated displacement profiles was 0.062 mm. This value reflects the variation associated with repeated scanning and data processing rather than specimen-to-specimen or build-to-build variability. Because only one specimen was fabricated for each configuration, manufacturing reproducibility could not be statistically evaluated, and the results are interpreted as controlled comparisons among the seven geometric configurations.

This procedure provided the experimental basis for comparing the distortion responses of structures with different geometric configurations after support removal.

## 3. Computational Simulation

### 3.1. Thermo-Mechanical FE Framework

To interpret the experimentally observed distortion behavior, a coupled thermo-mechanical FE model was established to simulate both the LPBF process and the subsequent support removal operation. A three-dimensional thermo-mechanical FE model was employed for the LPBF simulations [39,40]. Detailed descriptions of the numerical framework and its application to additive manufacturing have been reported in previous studies, where the model was validated through a series of in situ thermo-mechanical measurements [41,42].

The numerical analysis was performed using a staggered thermal-mechanical scheme. At each time increment, the transient temperature field was first calculated and subsequently transferred to the mechanical analysis. This sequential coupling reduces the computational cost but neglects the feedback of mechanical deformation on the thermal field.

Progressive material deposition was represented using layerwise element activation. An equivalent thermal boundary condition was applied to account for the insulating effect of the surrounding powder bed, whereas the loose powder was excluded from the mechanical domain because its stiffness is negligible relative to that of the consolidated material. Four physical layers were grouped into one numerical layer to improve computational efficiency. This treatment is intended to retain the structural-scale thermal and mechanical response, although local scan-track-scale temperature and stress variations may be smoothed.

The WEDM stage was represented by deactivating the elements connecting the supports to the substrate. This idealization reproduces the change in structural constraint after cutting but does not resolve the transient electromechanical and progressive material-removal processes involved in WEDM.

### 3.2. Governing Thermo-Mechanical Equations

The transient temperature evolution during LPBF is governed by the conservation of energy:

(1) $$\dot{H}=-\nabla \cdot q+\dot{Q}$$

where $\dot{H}$ is the enthalpy rate and $\dot{Q}$ is the internal volumetric heat source, defined as:

(2) $$\dot{Q}=\frac{\dot{P}\eta }{V_{pool}}$$

where $\dot{P}$ is the nominal laser power, η is the absorption parameter and $V_{pool}$ denotes the melt-pool volume.

Heat conduction inside the material follows Fourier’s law:

(3) q=−k∇T

where k(T) is the thermal conductivity coefficient and ∇T is the thermal gradients.

Convective heat losses from the free surface are respectively described by:

(4) $$q_{conv}=h_{conv}(T-T_{room})$$

where $h_{conv}$ is the convective Heat Transfer Coefficient (HTC), T is the temperature of the part surface and $T_{room}$ is the room temperature.

Radiative heat loss is defined by the Stefan-Boltzmann law:

(5) $$q_{rad}=\varepsilon_{rad}\sigma_{rad}\left(T^{4}-T_{room}^{4}\right)$$

where $\varepsilon_{rad}$ is the emissivity coefficient and $\sigma_{rad}$ represents the Stefan Boltzmann constant.

The mechanical response is governed by static equilibrium and volumetric continuity:

(6) ∇·s+∇p+b=0

(7) $$\left(\nabla \cdot u-e^{T}\right)-\frac{p}{K}=0$$

where u is the displacement vector, b denotes body forces, $e^{T}$ represents thermal strain, and K is the temperature-dependent bulk modulus.

The Cauchy stress tensor is decomposed into hydrostatic and deviatoric components:

(8) $$\sigma =pI+s\left(u\right)$$

This formulation is used to represent volumetric stress buildup and deviatoric stress redistribution during cooling and support removal.

### 3.3. FE Mesh, Material Properties and Boundary Conditions

**Figure 4:**
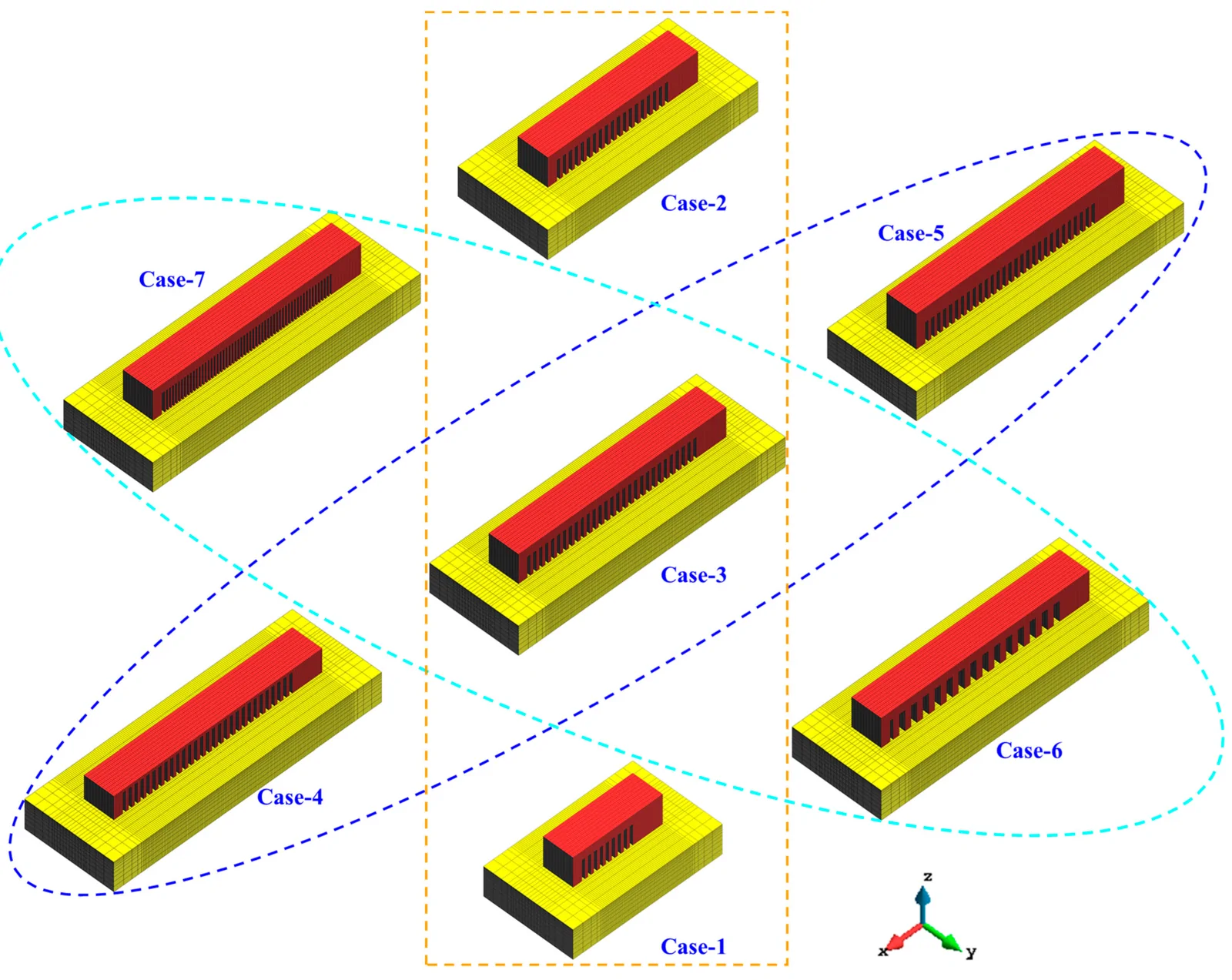
FE mesh models of the LPBF fabricated cantilever structures. The orange dashed square, blue dashed ellipse and cyan dashed ellipse denote groups with varying cantilever length, thickness and support density, respectively.

Figure 4 shows the FE models of the LPBF-fabricated cantilever structures and their corresponding meshes generated using the pre- and post-processing software *GiD* (v14) [42]. The numbers of 8-node Q1P0 hexahedral elements and nodes for each case are listed in Table 3. As the structural size increases, the computational cost rises rapidly if a fully resolved scanning strategy is adopted. To balance accuracy and efficiency, a layerwise activation scheme was used in the present simulations.

Accurate prediction of thermo-mechanical behavior during LPBF requires reliable temperature-dependent material data. The thermophysical and mechanical properties used in the simulations are summarized in Table 4 and Table 5 for TA15 and TC4 titanium alloys, respectively. For TA15, the density was assumed to be constant at 4450 kg/m^3^ over the temperature range considered, whereas the thermal conductivity, specific heat capacity, thermal expansion coefficient, elastic modulus, and elastic limit were defined as temperature-dependent. These properties were used to describe heat transfer, thermal strain accumulation, and stiffness degradation during the repeated heating and cooling cycles of the LPBF process.

Convective and radiative heat losses were applied to all exposed external surfaces of the substrate and printed part. For TA15, the emissivity was set to 0.7 and the convective heat transfer coefficient was taken as 10 W/(m^2^·°C). To account for heat dissipation through the contact between the substrate and the fixture, an enhanced heat transfer coefficient of 1000 W/(m^2^·°C) was assigned to the clamped end of the substrate. The ambient temperature was set to 20 °C throughout the simulation.

The mechanical boundary conditions were defined to be consistent with the LPBF experiments. A fully fixed constraint was applied to the lower surface of the substrate during the building stage. During the support removal simulation, the elements connecting the supports to the substrate were deactivated to reproduce the cutting operation, while constraints were retained at the bottom corners of the substrate to ensure numerical stability. In addition, because the support region contains partially activated material during the building process, the effective laser energy input in the support section was adjusted according to the corresponding area ratio.

The model is intended for structural-scale prediction of thermal history, stress evolution, and final distortion, rather than for resolving melt-pool- or scan-track-scale phenomena. Melt-pool hydrodynamics, individual scan-track geometry, powder-particle mechanics, and the transient electromechanical details of WEDM were not explicitly considered. Support removal was idealized as instantaneous element deactivation at the support-substrate interface. Consequently, track-scale temperature peaks, local stress concentrations around individual support legs, and transient stresses associated with progressive cutting may be smoothed or underestimated. The experimentally validated output is the post-cut structural warpage, while the simulated local stress fields and stress-redistribution paths are used primarily for mechanism interpretation.

## 4. Results and Discussion

### 4.1. Warpage Measurement and Model Validation

**Figure 5:**
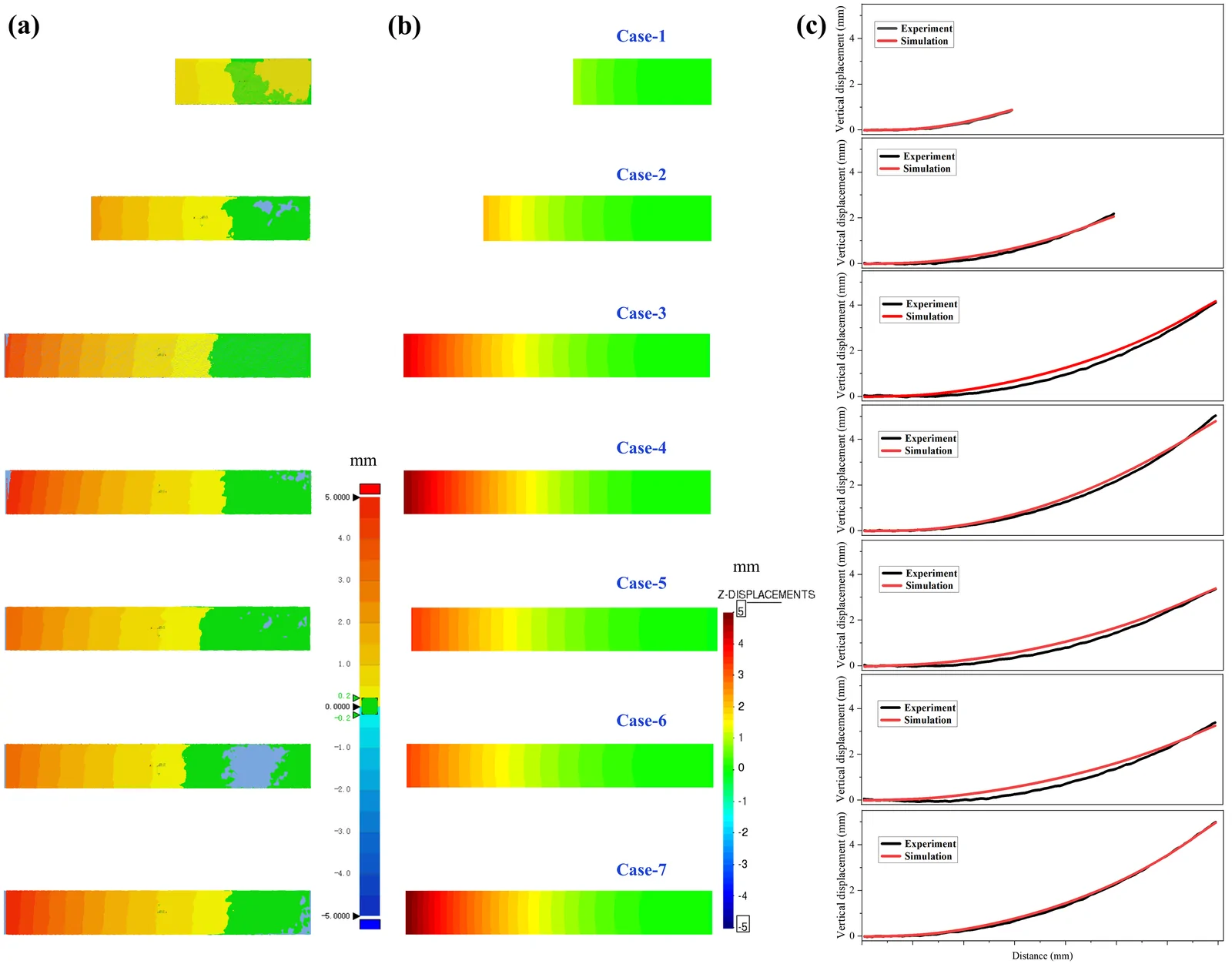
Comparison between measured and simulated warpage of cantilever structures: (**a**) 3D-scanned displacement contours; (**b**) simulated displacement contours; (**c**) vertical displacement distributions along the mid-line on the top surface.

As shown in Figure 5, the proposed thermo-mechanical FE model was validated by comparing the simulated warpage with the experimentally measured deformation of the fabricated cantilever structures. The out-of-plane displacement on the top surface after support removal was characterized by 3D scanning and directly compared with the numerical results.

Figure 5a,b show the measured and simulated displacement contours, respectively, while Figure 5c presents the corresponding vertical displacement distributions along the mid-line of the top surface. The simulation agrees well with the experimental results in both deformation pattern and warpage magnitude. The model successfully reproduces the upward bending behavior of all specimens and the variation in deformation with geometric configuration.

From the line profiles in Figure 5c, both the experimental and simulated results show a similar increase in vertical displacement from the fixed end to the free end, with the maximum deformation located near the free end. Although slight deviations exist in some local regions, the overall trends and peak values remain consistent. These differences are considered acceptable and may be attributed to process fluctuations, geometric imperfections, and simplifications in the numerical model.

The agreement between the measured and simulated deformation indicates that the model captures the overall distortion mode, the increase in displacement from the retained end to the free end, and the geometry-dependent ranking of warpage. The model is therefore used for the subsequent structural-scale interpretation of stress evolution and redistribution. Because the experimental validation is based on post-cut deformation rather than direct stress measurements, the predicted local stress magnitudes and peak positions should be interpreted with reference to the simplifying assumptions described above.

For quantitative validation, the measured and simulated vertical displacements were compared at corresponding positions along the mid-line of the cantilever top surface. The number of comparison points, mean absolute error (MAE), root mean square error (RMSE), and coefficient of determination (R^2^) for each configuration are summarized in Table 6. Across the seven configurations, the MAE ranges from 0.0260 to 0.1917 mm, while the RMSE ranges from 0.0330 to 0.2211 mm. The R^2^ values range from 0.9852 to 0.9993, indicating close agreement in the spatial variation in the measured and simulated deformation profiles. Cases 3 and 6 exhibit relatively larger absolute errors, whereas Cases 1 and 7 show the lowest MAE and RMSE values. Nevertheless, the combined contour, profile, and error-metric comparisons indicate that the model reproduces the overall post-cut warpage pattern and the geometry-dependent deformation trend. The validation applies to the structural-scale deformation response, while the predicted local stress peaks are used primarily for mechanism interpretation.

### 4.2. Effect of Cantilever Span

**Figure 6:**
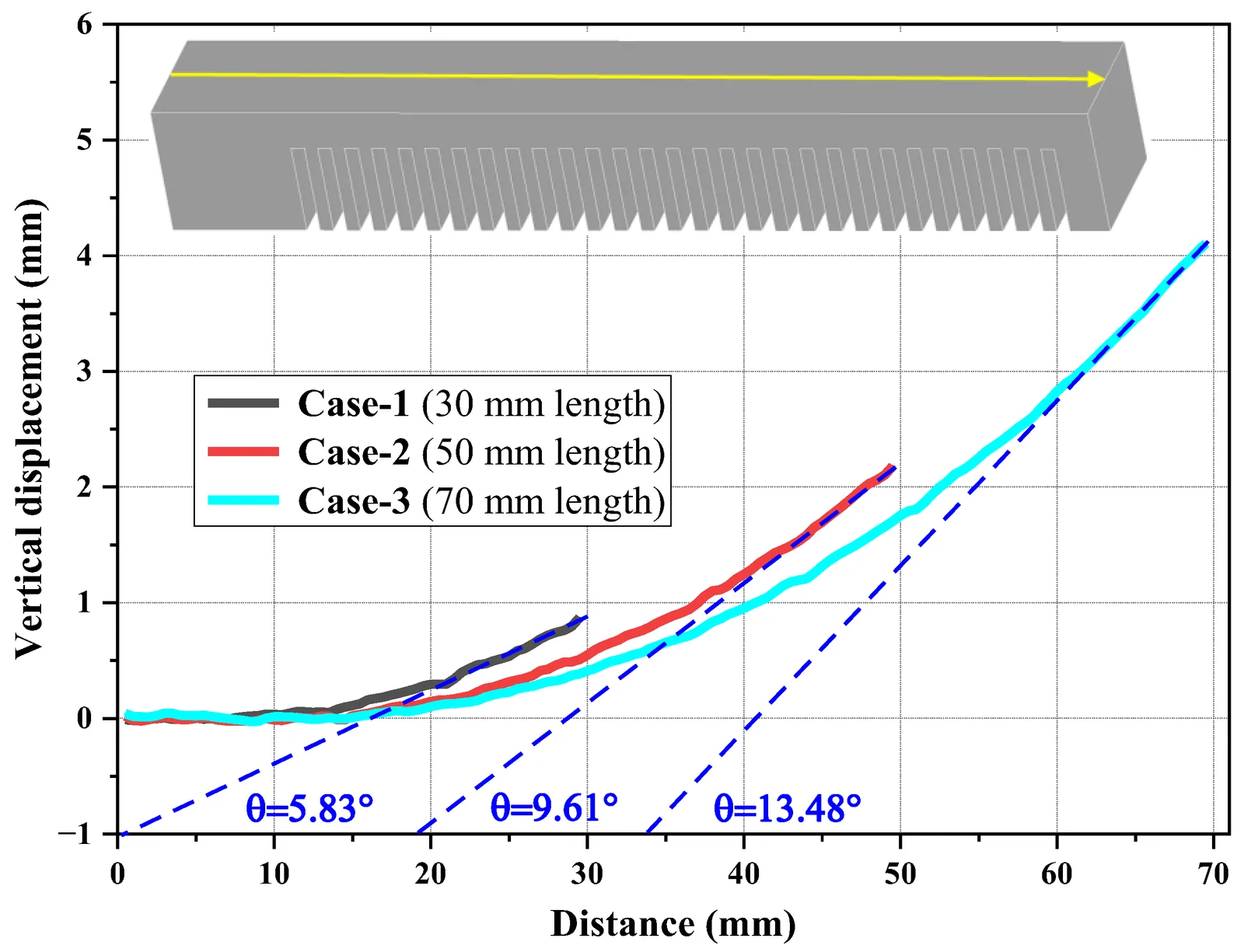
Measured vertical displacement profiles along the mid-line of the top surface (indicated by the yellow arrow) for cantilever structures with spans of 30, 50, and 70 mm, together with the corresponding curling angles after support removal.

As shown in Figure 6, the measured warpage increases progressively with cantilever span. Case-1 with a span of 30 mm exhibits the smallest deformation, whereas Case-3 with a span of 70 mm shows the largest. The curling angle correspondingly increases from 5.83° to 9.61° and 13.48°, indicating a progressively stronger bending response with increasing span. Because all specimens were fabricated using identical nominal processing parameters, differences in the measured warpage were not introduced by changes in the prescribed process settings. However, the local thermal and stress histories remained geometry-dependent. The span-dependent deformation is therefore interpreted through the combined effects of simulated stress redistribution, global flexural resistance, and the effective bending arm.

**Figure 7:**
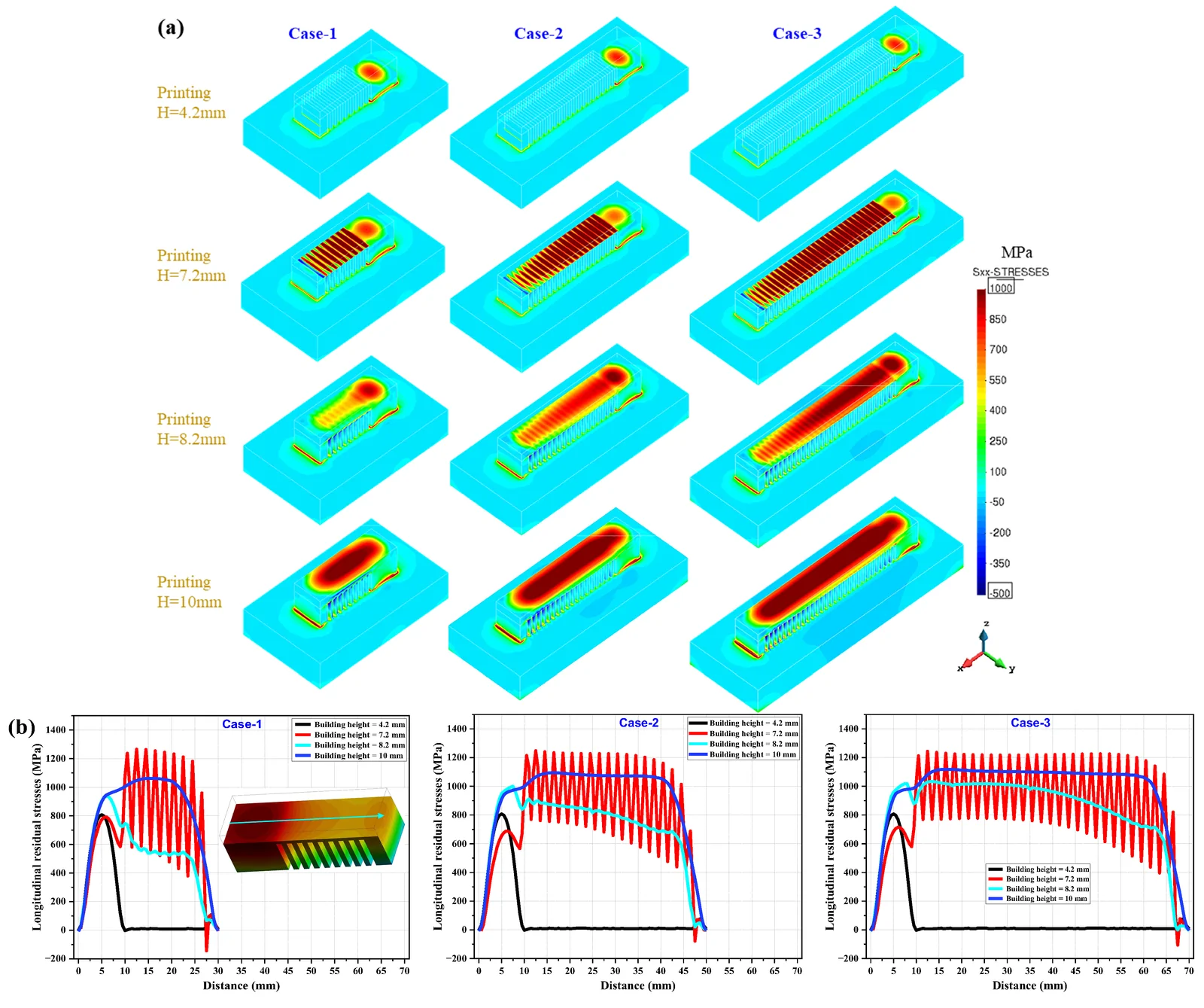
Evolution of longitudinal stress (*σ*_xx_) in cantilever structures with different spans during printing: (**a**) stress fields at building heights of 4.2, 7.2, 8.2, and 10 mm; (**b**) corresponding stress distributions along the mid-line of the top surface.

The longitudinal stress evolution in Figure 7 further clarifies the role of span during printing. At the early stage, when only the support region is deposited, the longitudinal stress remains at a relatively low level in all cases. Once the cantilever starts to form at H = 7.2 mm, pronounced stress fluctuations appear on the top surface, and Case-1 shows the strongest fluctuation. As the build height further increases, the stress fluctuation gradually decreases in all cases, while the effect of span becomes more evident. As the cantilever span increases, the larger suspended region develops a geometry-dependent thermal and mechanical response, leading to the simulated stress trend of Case-1 < Case-2 < Case-3 during the intermediate building stage. After deposition of the final layer; however, all cases approach similarly high simulated surface tensile-stress plateaus under the fully constrained as-built condition. This convergence suggests that the common support–substrate constraint contributes strongly to the final surface-stress state.

**Figure 8:**
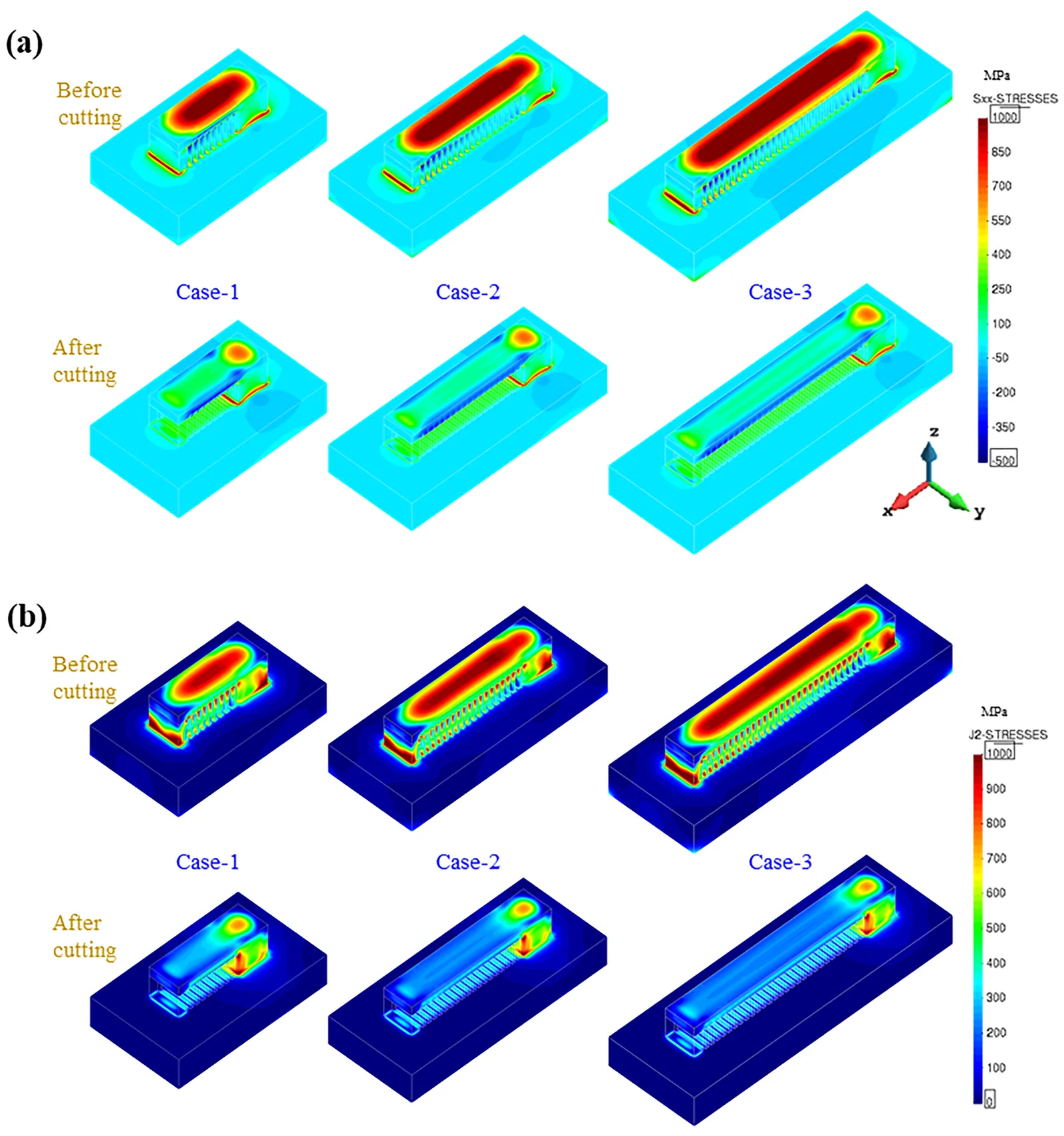
Stress redistribution in cantilever structures with different spans before and after support removal: (**a**) longitudinal stress (*σ*_xx_) fields; (**b**) von Mises stress fields.

**Figure 9:**
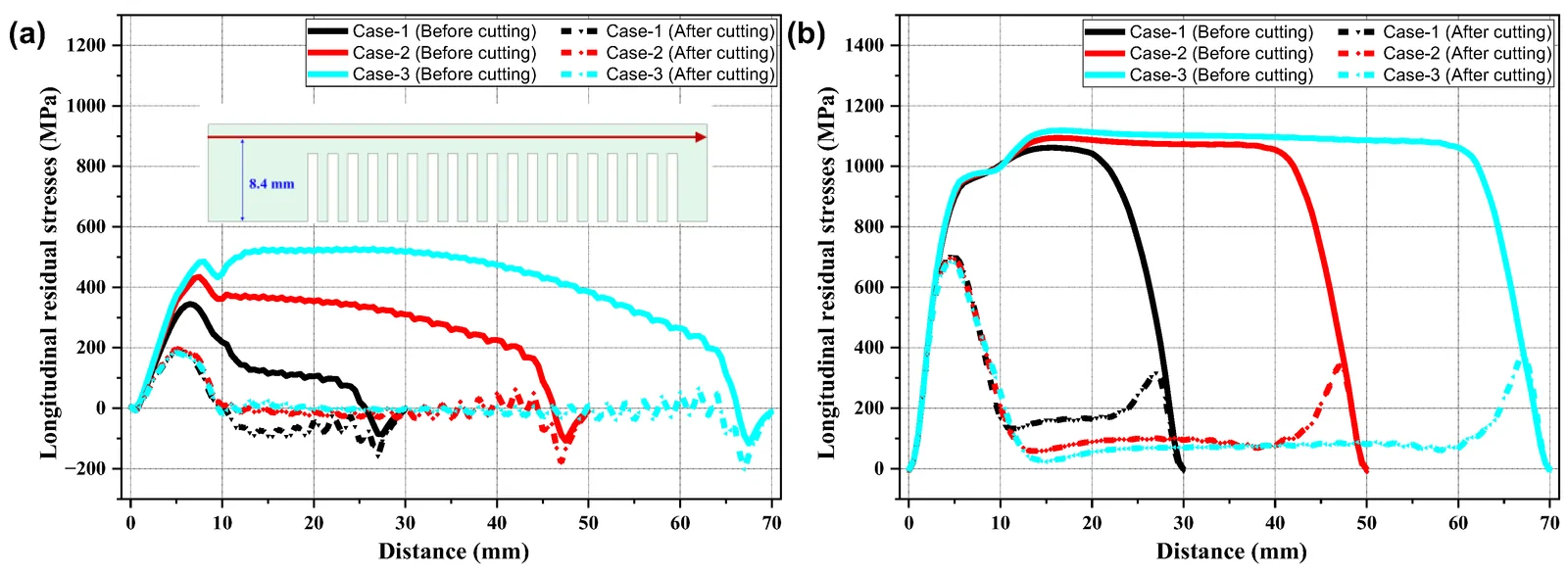
Redistribution of longitudinal stress (*σ*_xx_) in cantilever structures with different spans before and after support removal, extracted along two characteristic paths: (**a**) along the mid-line at a build height of 8.4 mm (indicated by the red arrow in the inset); (**b**) along the mid-line of the top surface.

Figure 8 and Figure 9 show the stress redistribution before and after support removal. Before cutting, all cases maintain a high tensile stress plateau because the cantilever is continuously constrained by the supports and substrate. After cutting, the stress distribution changes into a typical double-peak pattern, consisting of a high-stress root region, a relatively relaxed middle span, and a secondary peak near the free end associated with the terminal support region. This redistribution reflects the release of geometric constraint and the subsequent elastic recovery of the cantilever. More importantly, the residual stress level in the middle span is not directly proportional to the final deformation. Although Case-1 still retains relatively high residual stress in part of the span after cutting, its warpage remains the smallest because its higher global stiffness and shorter bending arm limit the extent to which stress redistribution is manifested as bending deformation. In contrast, Case-3 is more sensitive to stress release and therefore exhibits much larger upward bending.

Previous studies have established the sensitivity of LPBF cantilever deflection to geometry and processing conditions [10,11,19,24]. The present work extends this understanding by resolving the associated stress-redistribution process. Although the three spans approach similarly high simulated surface tensile-stress plateaus in the fully constrained state, their responses diverge after support removal because span changes both global flexural resistance and the effective bending arm. The contribution therefore lies not in restating the classical length-stiffness relationship, but in showing why comparable simulated as-built surface-stress levels can produce markedly different post-release distortion.

### 4.3. Effect of Cantilever Thickness

**Figure 10:**
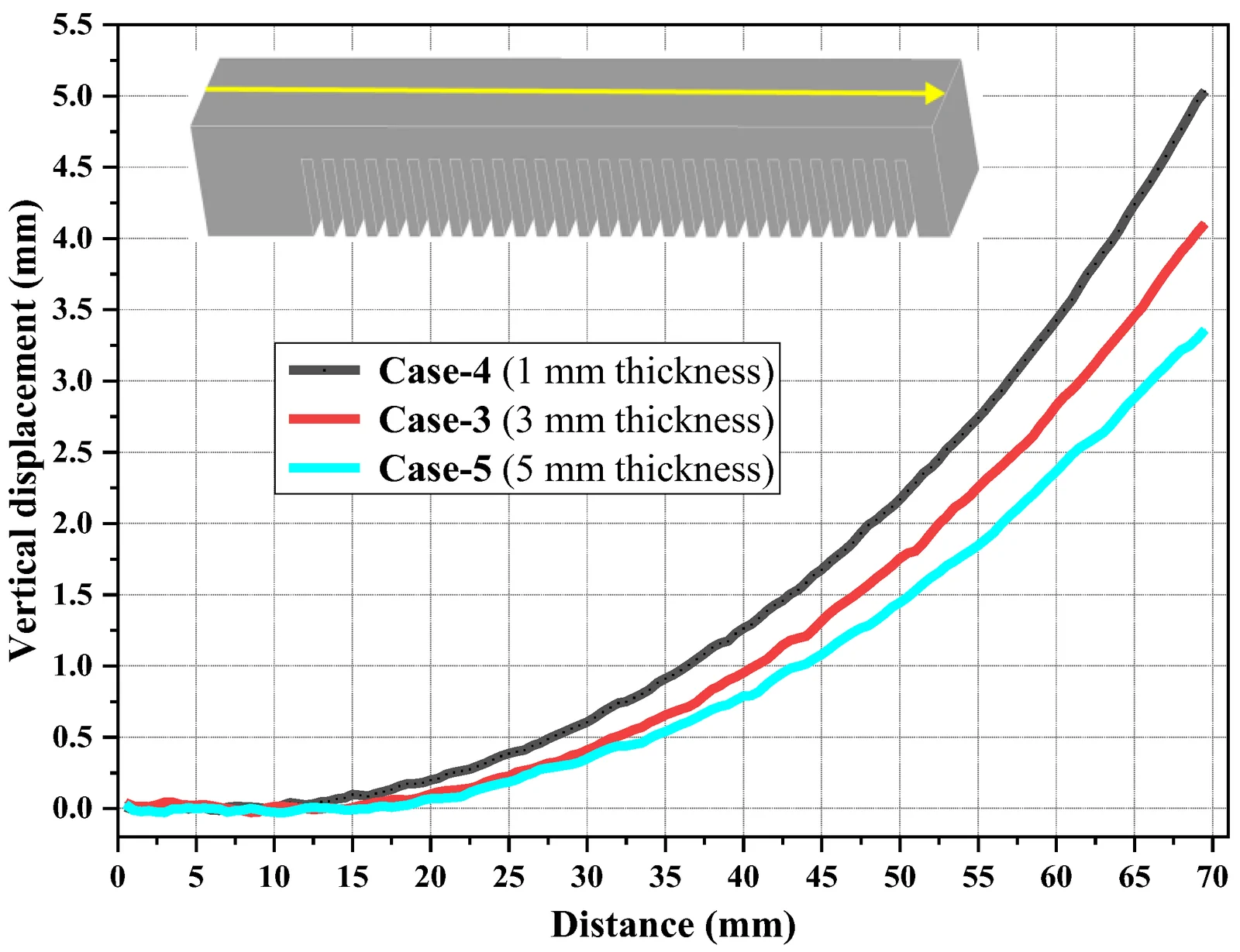
Measured vertical displacement profiles along the mid-line of the top surface (indicated by the yellow arrow) for cantilever structures with different thicknesses.

As shown in Figure 10, cantilever thickness is inversely correlated with deformation. Case-4 with a thickness of 1 mm exhibits the largest warpage, whereas Case-5 with a thickness of 5 mm shows the smallest deformation. All thickness cases were fabricated using identical nominal processing parameters, while their local thermal and mechanical responses remained geometry-dependent. The measured differences in post-cut warpage are therefore interpreted through the combined effects of thickness-dependent thermal response, sectional rigidity, and simulated stress redistribution.

**Figure 11:**
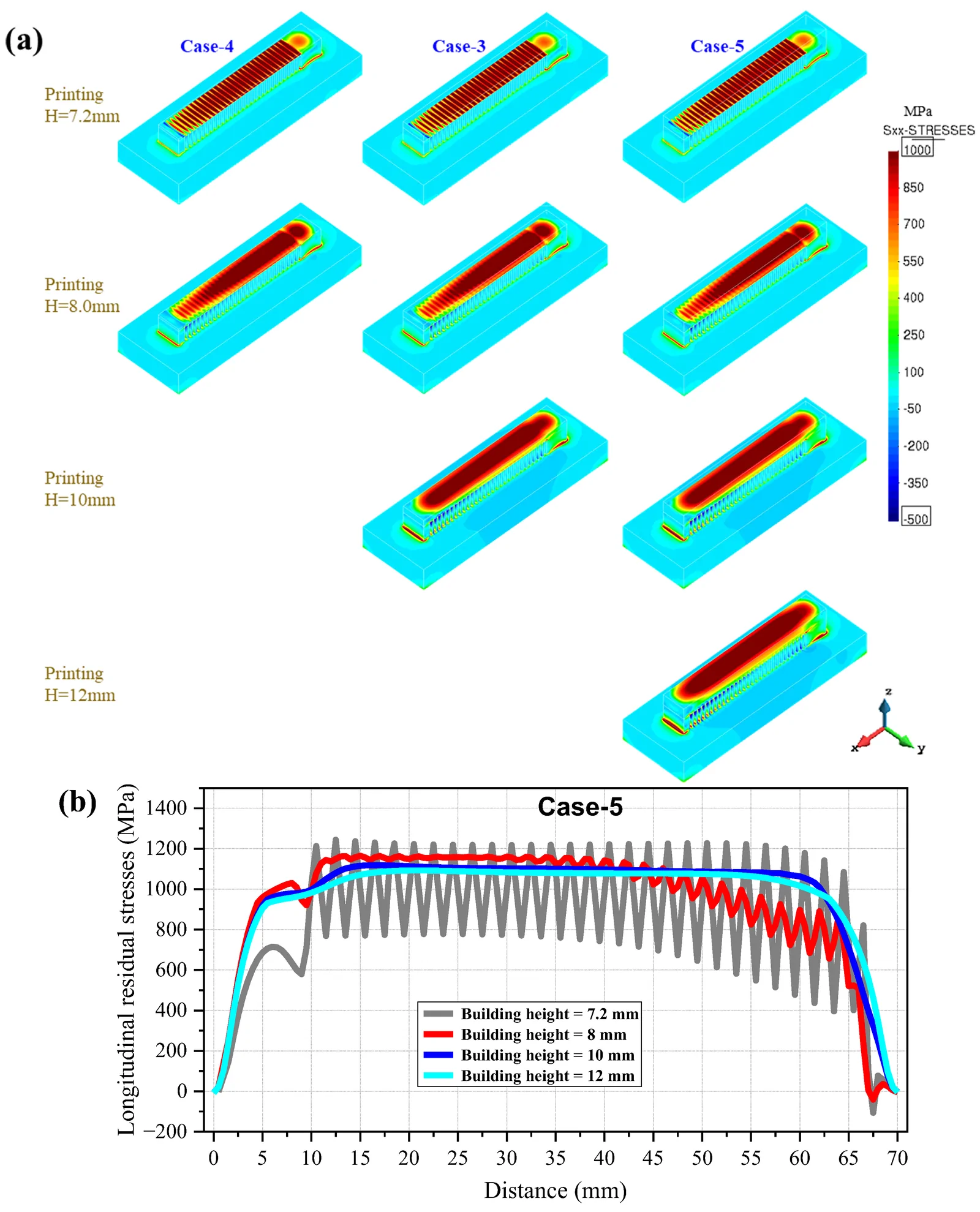
Evolution of longitudinal stress (*σ*_xx_) in LPBF cantilever structures with different thicknesses at selected building heights: (**a**) stress fields for Case-4, Case-3, and Case-5; (**b**) longitudinal stress profiles of Case-5 extracted along the mid-line of the top surface.

The longitudinal stress evolution in Figure 11 further reveals the role of thickness during printing. At the early stage of deposition, the thinner cantilever shows stronger stress fluctuation, indicating higher sensitivity to thermal cycling and local elastic deflection. As thickness increases, the stress field becomes more continuous and stable. This is because a thicker cantilever provides both greater thermal inertia and higher sectional stiffness, which suppress local deformation during deposition and promote the retention of thermal strain in the form of residual stress. As a result, although the final surface stress levels are similar under the fully constrained as-built condition, the deformation tendency remains markedly different.

**Figure 12:**
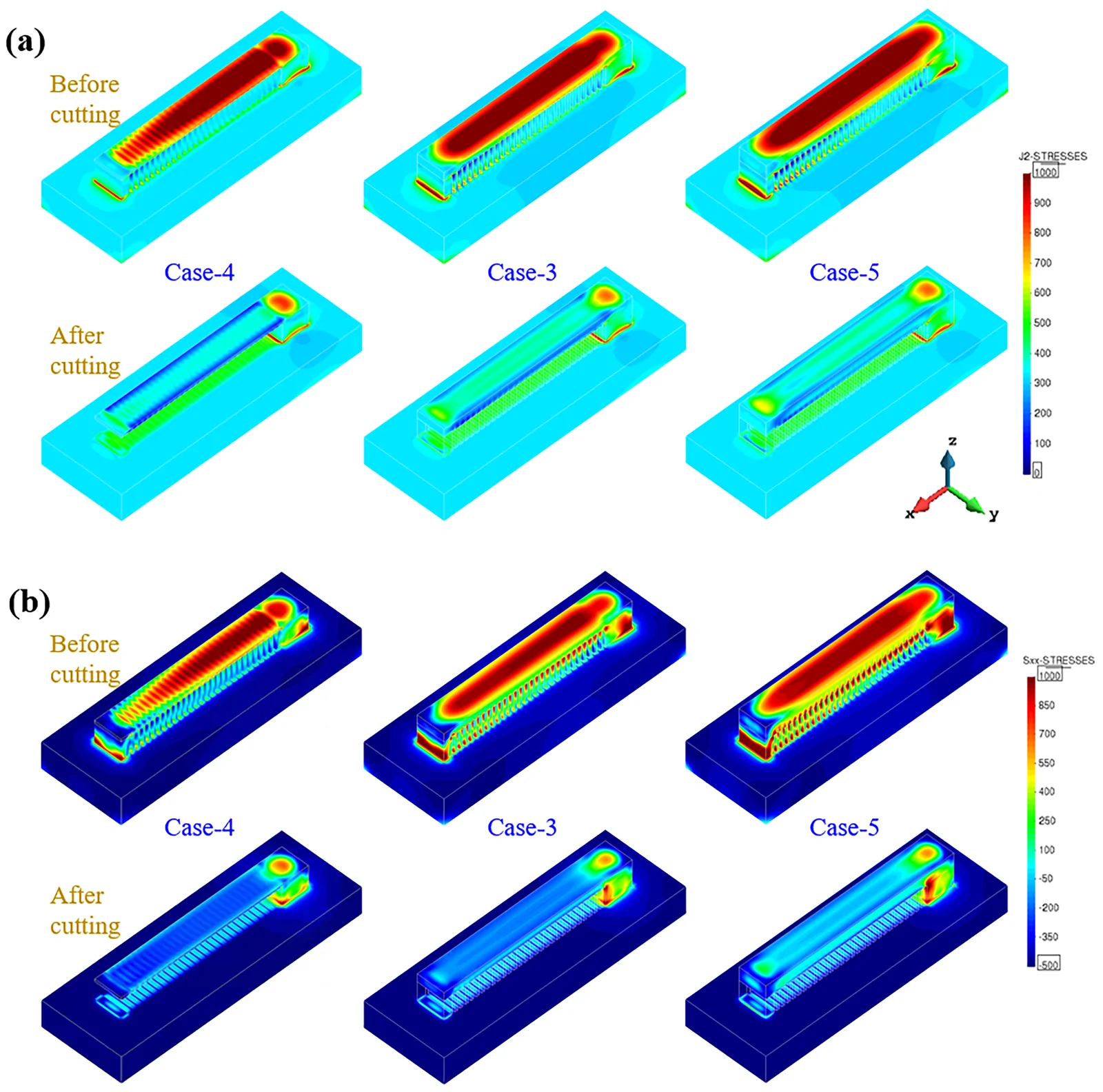
Stress redistribution in cantilever structures with different thicknesses before and after support removal: (**a**) longitudinal stress (*σ*_xx_) fields; (**b**) von Mises stress fields.

**Figure 13:**
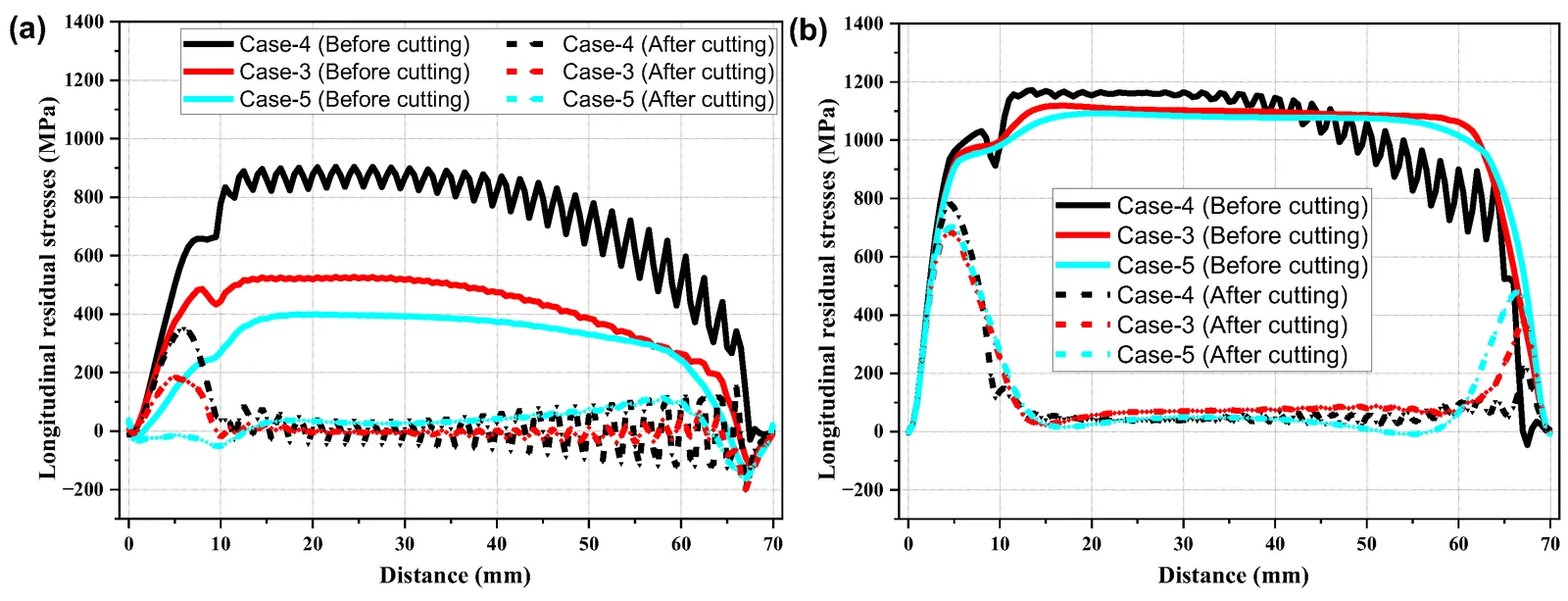
Redistribution of longitudinal stress (*σ*_xx_) in cantilever structures with different thicknesses before and after support removal, extracted along two characteristic paths: (**a**) along the mid-line at the mid-height of the cantilever; (**b**) along the mid-line of the top surface.

Figure 12 and Figure 13 show the stress redistribution before and after support removal. Figure 13a, extracted along the mid-line at the mid-height of the cantilever, highlights the difference in internal stress storage before cutting. Before support removal, Case-4 exhibits the highest stress level and the strongest fluctuation along this internal path, whereas the corresponding stress level decreases with increasing thickness. This indicates that the thin cantilever is more prone to repeated elastic accommodation during deposition, leading to a strongly non-uniform internal stress state. After cutting, the stress along this path is substantially relaxed in all cases, and the difference between the cases is greatly reduced. This behavior suggests that the stress stored in the interior of the thin cantilever is more readily released through elastic recovery once the support constraint is removed.

A different feature is observed in Figure 13b, which shows the stress distribution along the mid-line of the top surface. After cutting, all cases exhibit a root-dominated stress peak, a relatively relaxed span region, and a secondary peak near the free end. However, the redistribution pattern differs clearly with thickness. In Case-4, the root stress peak after cutting is the lowest, while the final warpage is the largest. This indicates that the thin cantilever undergoes more extensive stress release and bending recovery after support removal. Owing to its low flexural rigidity and greater deformation compatibility, the stress redistribution is manifested as a larger upward bending response rather than as a relatively high retained local stress. By contrast, Case-5 shows a more uniform stress field before cutting and substantial stress relaxation in the middle span after cutting. It is therefore not accurate to state that Case-5 undergoes the least stress release. Its characteristic feature is that relatively high residual stress is still retained near the free end, while the overall deformation remains small because the high sectional stiffness strongly suppresses curvature development.

Figure 13 also shows that the free-end stress peak shifts leftward with increasing thickness. This indicates that the active stress redistribution zone is not fixed at the geometric end, but depends on the balance between local stiffness, stress relaxation, and boundary effects. As thickness increases, the higher stiffness suppresses local curvature at the terminal region and causes the stress redistribution zone to extend further inward.

The inverse relationship between thickness and warpage is consistent with established flexural-rigidity arguments [23,26,27] and with previous additive-manufacturing studies reporting strong dimensional effects on residual distortion [19,24]. The present analysis further shows that simulated post-cut stress retention and final warpage do not necessarily vary in the same direction. The 1 mm cantilever exhibits the lowest simulated post-cut root-stress peak but the largest deformation, whereas the thicker cantilever retains localized high stress while developing limited curvature. These results indicate that the extent to which stress redistribution is manifested as structural bending depends strongly on sectional stiffness and cannot be inferred from the final local stress magnitude alone.

### 4.4. Effect of Support Density

**Figure 14:**
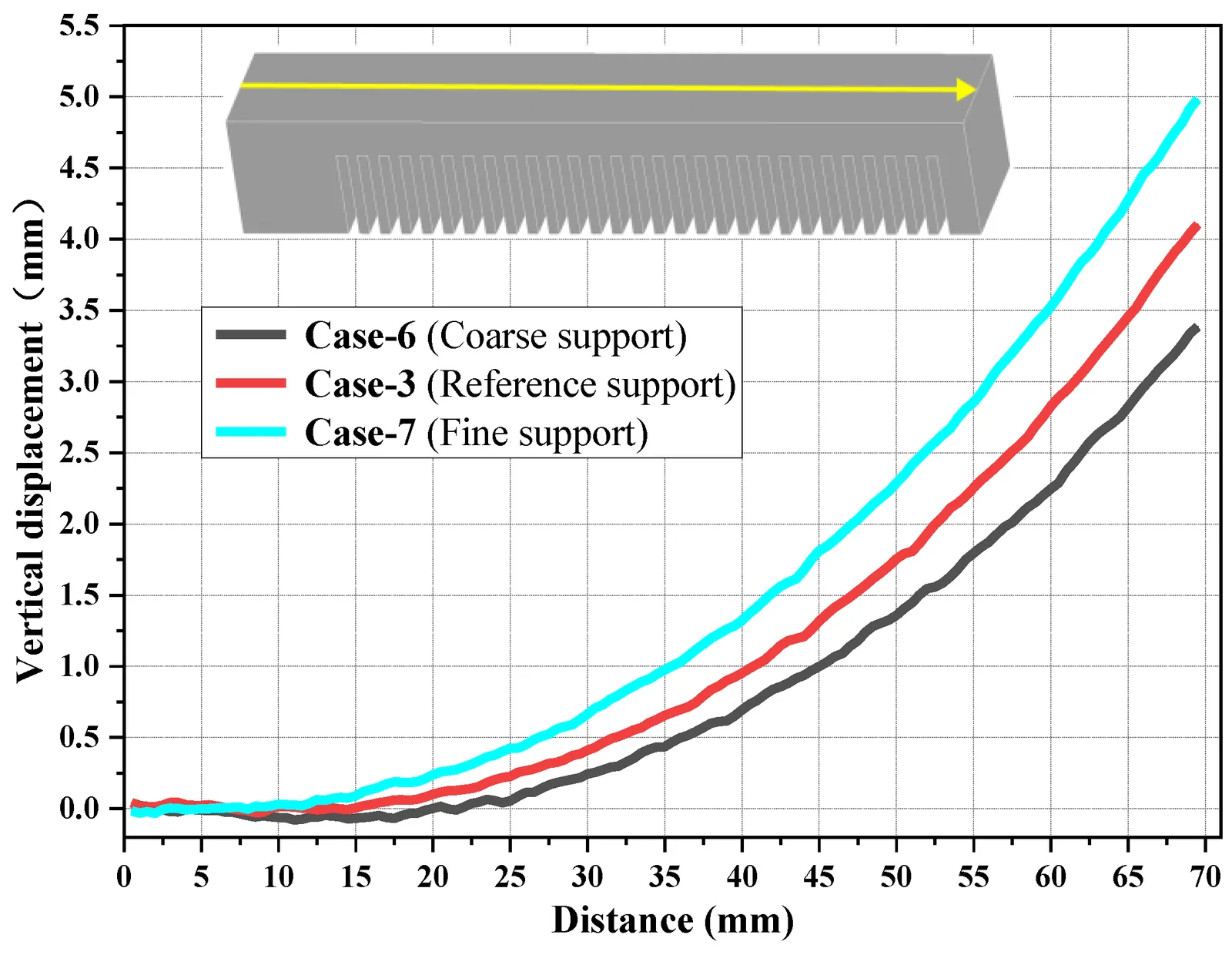
Measured vertical displacement profiles along the mid-line of the top surface (indicated by the yellow arrow) for cantilever structures with different support densities.

Figure 14 compares the measured deformation profiles of cantilever structures with different support densities. Case-6 with coarse supports shows the smallest deformation, whereas Case-7 with fine supports exhibits the largest warpage. Since the total support volume is kept constant, this difference is not caused by the amount of support material itself, but by the support geometry and the resulting change in local constraint and deformation compatibility beneath the cantilever.

The support-density effect involves competing mechanical and thermal factors. On the one hand, finer support legs have lower local stiffness, which is more favorable for local deformation and partial stress release. On the other hand, coarse supports with larger spacing create wider locally unsupported regions and a more heterogeneous local thermal and mechanical response, which may facilitate local stress relaxation during deposition. At the same time, the coarse support legs themselves provide stronger local restraint at the support-contact positions. The final deformation therefore depends on the combined effect of local support stiffness, support spacing, and the associated stress-release pathway.

**Figure 15:**
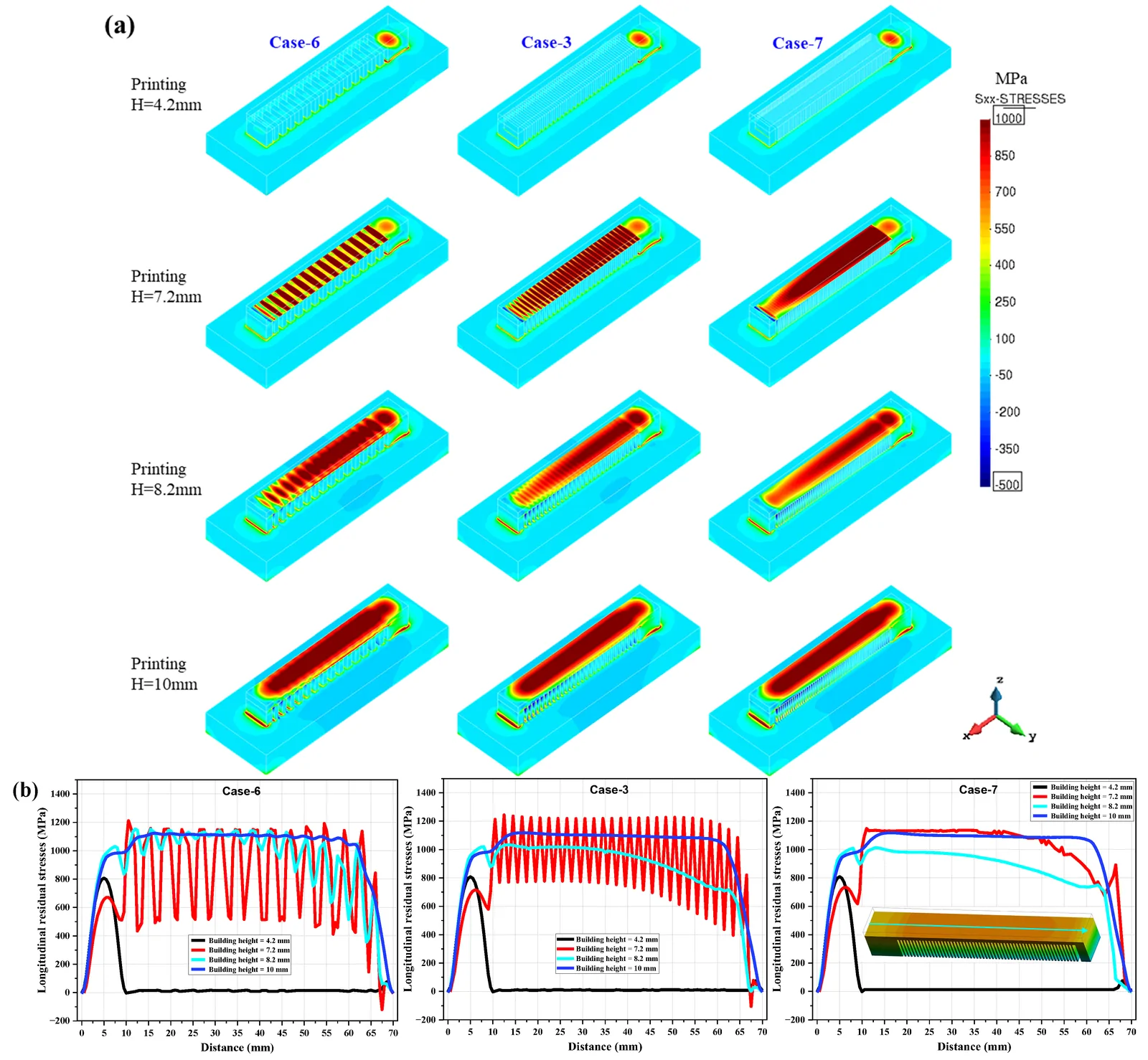
Evolution of longitudinal stress (*σ*_xx_) in cantilever structures with different support densities at selected building heights: (**a**) stress fields; (**b**) corresponding stress profiles along the mid-line of the top surface (indicated by the arrow in the inset), with curves of different colors corresponding to results at different building heights.

The stress evolution in Figure 15 reflects this competition. Case-6 exhibits stronger stress fluctuation during printing, especially at the early stage, indicating a more heterogeneous stress state associated with the discrete support layout. This suggests that the coarse-support configuration promotes stronger local variation in thermal and mechanical response, which is consistent with the presence of wider unsupported intervals between adjacent support legs. By contrast, Case-7 shows a more continuous tensile stress distribution along the cantilever, indicating that the fine-support layout leads to a more spatially uniform constraint condition at the structural scale, even though the local stiffness of each individual support leg is lower.

**Figure 16:**
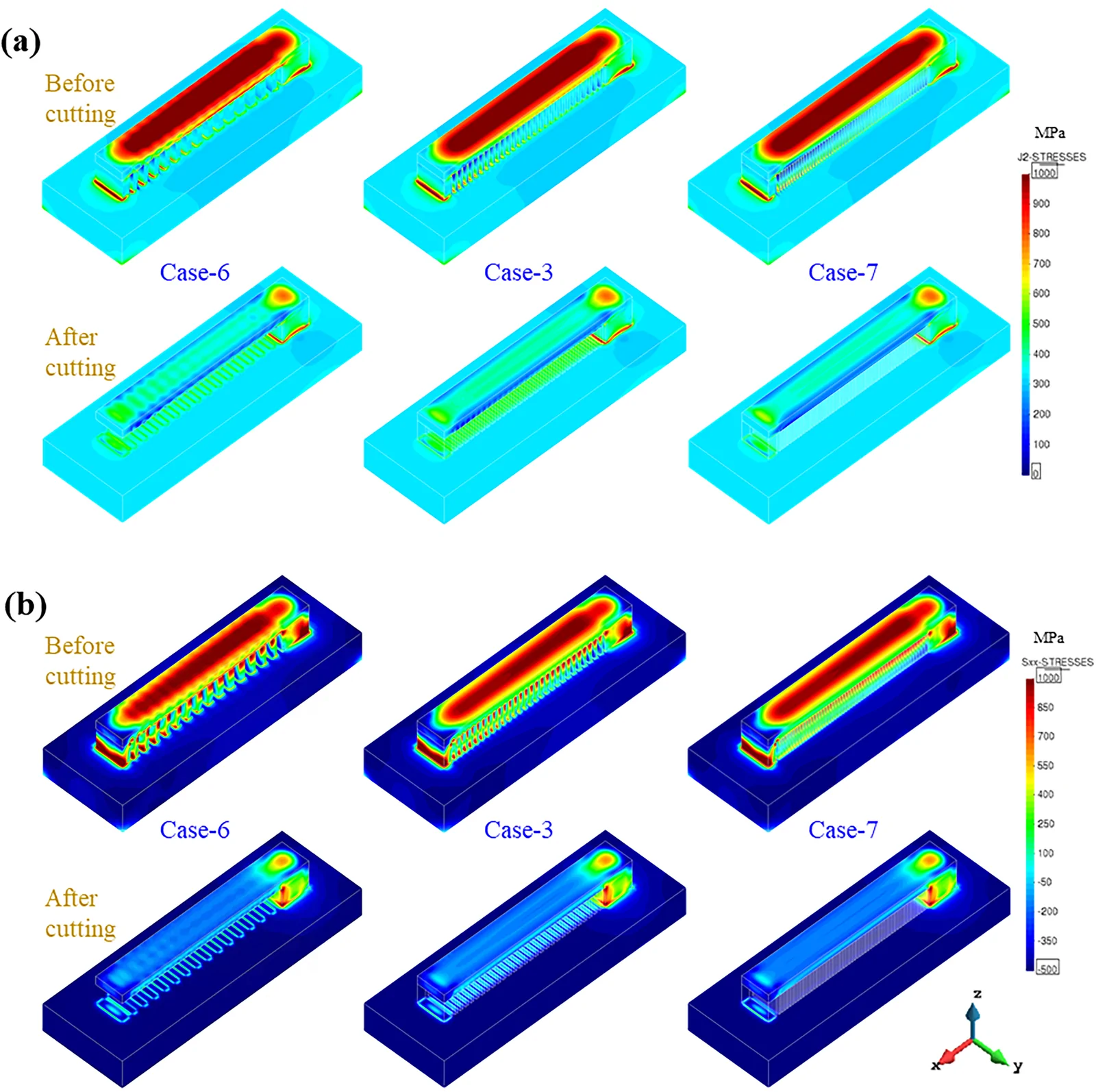
Stress redistribution in LPBF cantilever structures with different support densities before and after support removal: (**a**) longitudinal stress (*σ*_xx_) fields; (**b**) von Mises stress fields.

**Figure 17:**
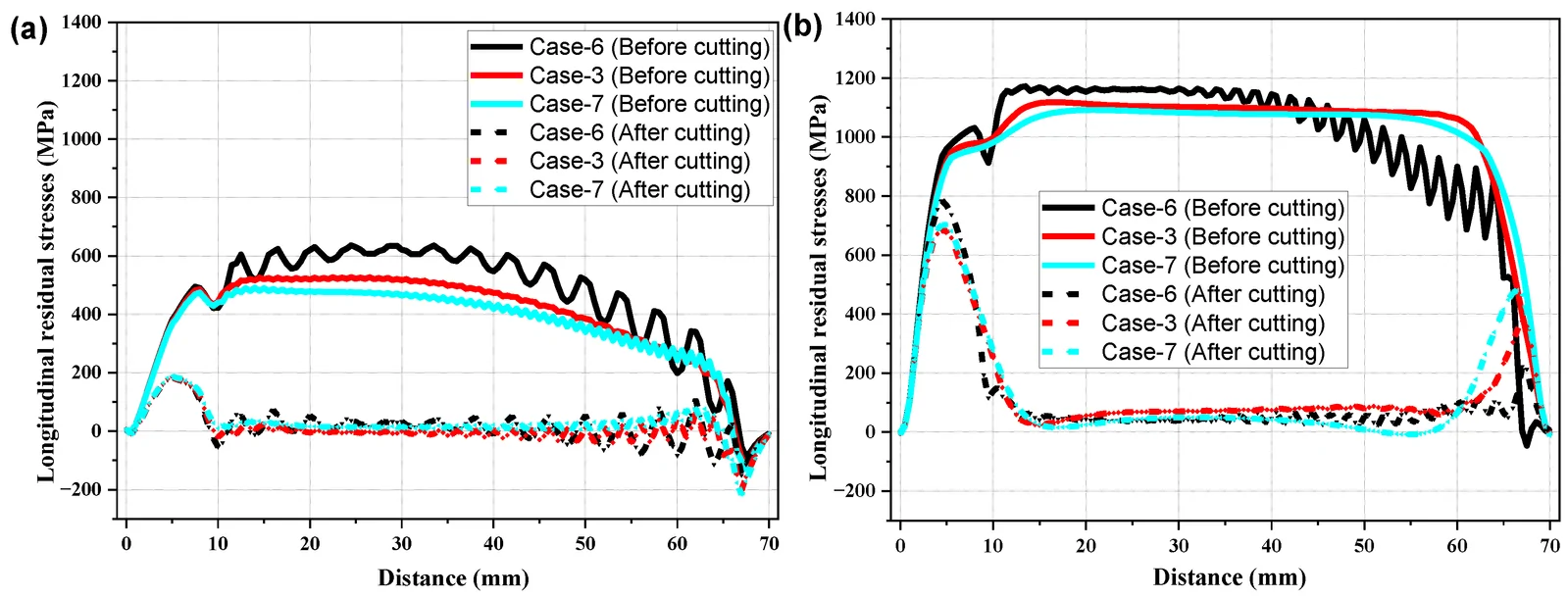
Redistribution of longitudinal stress (*σ*_xx_) in cantilever structures with different support densities before and after support removal, extracted along two characteristic paths: (**a**) along the mid-line at the mid-height of the cantilever; (**b**) along the mid-line of the top surface.

Figure 16 and Figure 17 further show the stress redistribution before and after support removal. After cutting, Case-6 exhibits stronger spatial stress fluctuation, reflecting the non-uniform stress storage associated with the discrete support pattern. Case-7, by comparison, retains a more continuous redistributed stress field and shows the largest final deformation. The simulated fields suggest that the fine-support configuration develops a more spatially continuous redistribution pattern at the structural scale, which is consistent with its larger measured bending response after support removal.

It should be noted that the layer-activation strategy is intended to capture structural-scale thermal and mechanical responses but does not fully resolve scan-track-scale thermal fluctuations or local stress concentrations around individual support legs and unsupported intervals. The support-density results should therefore be interpreted primarily in terms of the overall constraint mode, deformation compatibility, and post-removal warpage of the cantilever–support system. The predicted local stress values around individual support units are used as qualitative indicators rather than as fully resolved quantitative results.

Accordingly, the effect of support density is governed by the combined influence of support stiffness, support spacing, and deformation compatibility. Coarse supports generate stronger local restraint but also larger unsupported intervals that may facilitate local stress relaxation, whereas fine supports reduce the stiffness of individual support legs but provide a more spatially continuous support condition at the structural scale. The larger warpage observed in the fine-support case therefore reflects the macroscopic constraint and stress-release behavior of the cantilever-support system rather than the local stiffness of a single support leg alone.

Previous studies have shown that support configuration affects support strength, residual stress, distortion, and manufacturability [17,18,28], while many optimization and equivalent-property approaches emphasize material consumption, removal effort, or homogenized support behavior [35,36,37]. By holding the total support volume constant, the present comparison separates the influence of support distribution from that of support material amount. The results show that a larger number of finer supports does not necessarily reduce warpage, because the reduced stiffness of individual support legs is accompanied by a more spatially continuous system-level constraint. The resulting deformation therefore depends on the combined effects of support-leg stiffness, support spacing, local thermal and mechanical response, and deformation compatibility. This finding provides a controlled design comparison rather than a universal support-optimization rule.

## 5. Conclusions

This study combined three-dimensional scanning and thermo-mechanical FE modeling to investigate the post-cut distortion of LPBF cantilevers with different spans, thicknesses, and support configurations. The main conclusions are as follows:

(1) The measured post-cut warpage increased with cantilever span. Although the different spans approached similar simulated surface tensile-stress levels in the constrained as-built state, the longer cantilevers developed greater deformation after support removal because of their lower flexural resistance and larger effective bending arm.
(2) The measured post-cut warpage decreased with increasing cantilever thickness. The simulated stress paths showed stronger fluctuation and greater stress relaxation in the thin cantilever, whereas the thick cantilever retained relatively high localized stress while developing limited curvature because of its higher sectional rigidity.
(3) Under a constant total support volume, the fine-support configuration exhibited greater measured warpage than the coarse-support configuration. The simulated results indicate that this response arises from the combined effects of support-leg stiffness, support spacing, system-level constraint continuity, and deformation compatibility rather than from support volume alone.

## Figures and Tables

**Table 1:** Chemical compositions of the TA15 powder (wt.%) [38].

Element	Al	V	Zr	Mo	Fe	Si	C	O	N	H	Ti
wt.%	6.09	1.25	2.16	0.81	0.024	0.023	<0.01	0.073	0.0092	<0.0017	Bal.

Note: The chemical composition of the TA15 powder is consistent with that used in our previous published work on LPBF-fabricated TA15 alloy.

**Table 2:** Processing parameters used for the LPBF process.

Laser Power (W)	Layer Thickness (µm)	Scan Speed (mm/s)	Hatch Distance (µm)	Laser Beam Diameter (µm)	Scan Strategy	Scan Fill Mode
280	60	1250	100	100	Zigzag	10 mm wide strip

Note: The LPBF processing parameters are adopted from our previous systematic study on TA15 titanium alloy.

**Table 3:** Numbers of FE elements and nodes.

Samples	Structural Characteristics	Number of Hexahedral Elements	Number of Nodes
Case-1	30 mm length, 3 mm thickness, reference support	37,700	46,585
Case-2	50 mm length, 3 mm thickness, reference support	58,700	74,085
Case-3	70 mm length, 3 mm thickness, reference support	79,700	101,585
Case-4	70 mm length, 1 mm thickness, reference support	65,700	86,075
Case-5	70 mm length, 5 mm thickness, reference support	93,700	117,095
Case-6	70 mm length, 3 mm thickness, coarse support	79,700	101,585
Case-7	70 mm length, 3 mm thickness, fine support	79,700	112,057

**Table 4:** Temperature-dependent material properties of TA15 [38].

Temperature (°C)	Density (kg/m^3^)	Thermal Conductivity (W/(m·°C))	Heat Capacity (J/(kg·°C))	Poisson’s Ratio	Thermal Expansion Coefficient (μm/m/°C)	Young’s Modulus (GPa)	Elastic Limit (MPa)
20	4450	8	510	0.39	8.8	118	1014
100	4450	8.8	545	0.39	8.9	110	917
200	4450	10.2	587	0.39	9.0	100	843
300	4450	10.9	628	0.39	9.2	95	795
400	4450	12.2	670	0.39	9.7	90	754
500	4450	13.8	712	0.39	10.0	80	772
600	4450	15.1	755	0.39	10.4	73	622
700	4450	16.8	838	0.39	10.9	63	400
800	4450	18.0	880	0.39	11.0	54	350
900	4450	19.7	922	0.39	11.1	43	200

**Table 5:** Temperature-dependent material properties of TC4 [22].

Temperature (°C)	Density (kg/m^3^)	Thermal Conductivity (W/(m·°C))	Heat Capacity (J/(kg·°C))	Poisson’s Ratio	Thermal Expansion Coefficient (μm/m/°C)	Young’s Modulus (GPa)	Elastic Limit (MPa)
20	4420	7	546	0.345	8.78	110	850
205	4395	8.75	584	0.35	10	100	630
500	4350	12.6	651	0.37	11.2	76	470
995	4282	22.7	753	0.43	12.3	15	13
1100	4267	19.3	641	0.43	12.4	5	5
1200	4252	21	660	0.43	12.42	4	1
1600	4198	25.8	732	0.43	12.5	1	0.5
1650	3886	83.5	831	0.43	12.5	0.1	0.1
2000	3818	83.5	831	0.43	12.5	0.01	0.01

**Table 6:** Quantitative error metrics between measured and simulated post-cut displacement profiles.

Error Metrics	Case-1	Case-2	Case-3	Case-4	Case-5	Case-6	Case-7
Number of comparison points	59	99	139	139	139	139	139
MAE (mm)	0.0260	0.0724	0.1836	0.0992	0.1556	0.1917	0.0497
RMSE (mm)	0.0330	0.0848	0.2061	0.1212	0.1775	0.2211	0.0624
R^2^	0.9952	0.9926	0.9930	0.9959	0.9924	0.9852	0.9993

## Data Availability

The original contributions presented in this study are included in the article. Further inquiries can be directed to the corresponding author.
